# Oral health knowledge, attitudes, and practices across four communities in the Western Cape, South Africa: A cross-sectional study

**DOI:** 10.4102/jphia.v17i1.1687

**Published:** 2026-07-10

**Authors:** Talitha Crowley, Million Bimerew, Thabani Noncungu, Furaha Akimanimpaye, Rukshana Francis, Margaret Hoffman, Pelisa Khonco, Sergio Hendricks, Mogasweri Naidoo, John J. Musafiri, Penelope Martin, Jennifer Chipps

**Affiliations:** 1 School of Nursing, Faculty of Community and Health Sciences, University of the Western Cape, Cape Town, South Africa

**Keywords:** oral health, dental caries, periodontal disease, knowledge, attitudes, practices

## Abstract

**Background:**

Oral health conditions are preventable, but frequently neglected because of limited knowledge, negative attitudes, and misconceptions within communities.

**Aim:**

This study aimed to determine the oral health knowledge, attitudes, and practices and factors associated with these outcomes and self-reported oral pain among community members across four communities (two urban and two semi-rural) in the Western Cape, South Africa.

**Setting:**

Two urban and two semi-rural communities in the Western Cape, South Africa.

**Methods:**

A cross-sectional survey using a researcher-administered questionnaire with validated instruments (The World Health Organization [WHO] Oral Health Assessment and the Rustvold Scale) was conducted among 776 adults, with knowledge, attitudes, and practices measured using composite scores. Data were analysed using descriptive and multivariable analyses to identify socio-demographic factors associated with knowledge, attitudes, and practices, as well as factors associated with self-reported oral pain.

**Results:**

Most respondents were female (*n* = 567, 73.1%) with a mean age of 42.3 ± 14.2 years. Overall knowledge was moderate (mean score: 68.4%, 6.84, ± 1.4). While most respondents reported daily toothbrushing and using fluoride toothpaste, only 13.8% used dental floss, and fewer than 20% had visited a dentist in the past year, primarily for pain. In multivariable logistic regression analysis, semi-rural residence (adjusted odds ratio [aOR]: 2.1; 95% confidence interval [CI]: 1.4–3.1), female gender (aOR: 1.6; 95% CI: 1.2–2.3), and poor self-reported health status aOR: 1.8; 95% CI: 1.2–2.7 were associated with higher odds of self-reported oral pain.

**Conclusion:**

Despite moderate knowledge and positive attitudes, gaps in oral health practices persist, with higher odds of oral pain among semi-rural residents and those with poorer health.

**Contribution:**

Findings support targeted oral health promotion and improved access to preventive care in semi-rural communities.

## Introduction

The World Health Organization’s (WHO) Global Oral Health Programme (GOHP) has played a key role in raising global awareness of the importance of oral health, recognising its critical contribution to overall health and quality of life. Despite these efforts, oral diseases remain a significant public health issue even in high-income countries, affecting over 3.5 billion people worldwide.^1^ This burden continues to rise in many low-income and middle-income countries. Oral diseases such as dental caries, periodontal disease, and oral cancers contribute to pain, discomfort, disfigurement, and even death in severe cases.^2^ Inequities in access to preventive care, treatment services, and oral health education further contribute to this global burden, underscoring the urgent need for integrated strategies that address the social, economic, and environmental factors that may influence oral health.^3,4^

Oral health is a fundamental aspect of overall well-being, influencing an individual’s ability to eat, speak, and maintain social interactions. It affects quality of life, self-esteem, and overall health outcomes.^5^ Oral health knowledge, attitudes, and practices are related to oral health outcomes and overall well-being.^6^ Inadequate oral health knowledge, negative attitudes, and poor oral health practices can increase the risk of oral diseases, such as dental caries and periodontal disease.^6^ Globally, studies have examined oral health knowledge, attitudes, and practices across diverse populations, including individuals with chronic diseases, students, parents, and teachers.^6,7,8,9^ In China, Wang et al. found high levels of knowledge, positive attitudes, and commendable practices among parents and kindergarten teachers, with daily habits directly influenced by knowledge and attitudes.^9^

A lack of oral health literacy has been associated with oral health conditions, such as dental caries and periodontal disease.^10^ Awareness of oral health is crucial for establishing healthy habits, as good knowledge has been linked to better oral health outcomes. Individuals with an understanding of diseases and their causes are more likely to adopt health-related behaviours, particularly when they feel empowered to take control of their own health.^11^ Individuals often lack basic knowledge about oral health, including proper oral hygiene practices, the importance of regular dental check-ups, and the relationship between oral health and general health.^12^ An oral health knowledge, attitudes, and practices study among healthcare workers in India found that some healthcare workers were unaware of the link between oral health and systemic health.^12^ These knowledge gaps are often because of socio-economic factors, educational background, and cultural beliefs, which can impact individuals’ access to oral health information and resources.^13^

Attitudes and beliefs towards oral health play a crucial role in shaping individuals’ behaviours and practices.^14^ Positive attitudes towards oral health, such as perceiving oral health as important for overall well-being and feeling confident in one’s ability to maintain good oral hygiene, are associated with better oral health outcomes.^15^ Conversely, negative attitudes and misconceptions, such as fear of dentists, beliefs in traditional remedies, or religious practices such as fasting, can lead to avoidance of dental care and inadequate oral hygiene practices.^16^

Research on oral health practices has identified disparities in preventive behaviours and dental care utilisation. Adherence to recommended oral hygiene practices, such as brushing teeth twice a day and regular flossing, varies among different populations.^17^ Factors such as age, level of education, and income level or socio-economic status influence individuals’ engagement in preventive oral health behaviours.^1^ Dental care utilisation also varies, with some individuals facing barriers such as financial constraints, a lack of access to dental services, and fear or anxiety associated with dental visits.^18^ A study among secondary-level students in Nepal, South Asia, found reluctance to visit a dentist and poor oral health practices, despite good knowledge and attitudes.^8^

South Africa has a high burden of oral health diseases. Smit et al. reported that the prevalence of oral caries among South African children aged 6 years was 84% in 2015, and among children aged 12 years, it was 67%.^19^ Chikte et al. conducted a cross-sectional study in Bellville, Western Cape, South Africa, and found that 68% of adults experienced high prevalence of decayed, missing, and filled teeth (DMFT) scores.^20^

The Western Cape province of South Africa encompasses diverse communities with varying socio-economic backgrounds, cultural practices, and access to healthcare. However, there is a lack of comprehensive information regarding oral health knowledge, attitudes, and practices within these communities. Assessing these factors is essential to identify existing gaps, misconceptions, and barriers that may contribute to poor oral health outcomes. Therefore, understanding community members’ knowledge, attitudes, and practices is crucial for designing effective interventions that promote oral health in a targeted manner. This research aimed to determine oral health knowledge, attitudes, and practices, as well as factors associated with these outcomes and self-reported oral pain, among community members in four communities in the Western Cape province.

## Research methods and design

### Study design

A quantitative descriptive survey with researcher-administered questionnaires was conducted, using trained data collectors who directly engaged with respondents to ask questions and record their responses. This method ensured greater consistency in data collection, minimised misunderstandings or misinterpretations of questions, and allowed for clarification as necessary.

### Research setting

The study was conducted in four communities within the Cape Town Metropole of the Western Cape province, South Africa, with a total population of 4 772 846.^21^ These communities are directly linked to the outreach community services of the University of the Western Cape. Site A is an urban central business community located approximately 20 km from Cape Town city centre. It is a busy area and a major transportation hub, situated next to a taxi rank and train stations connecting people from various parts of the Western Cape. Site B is a semi-rural area located 10 km northeast of Durbanville and approximately 40 km from Cape Town city centre. Site C is a semi-rural area situated 15 km north of Durbanville and around 53 km from Cape Town city centre. Site D is an urban area located 20 km from the Cape Town city centre. The three main languages in these areas are Afrikaans, English, and isiXhosa.

### Population and sampling

The target population comprised individuals within the selected geographic areas whose prevalence of oral health problems is unknown or undocumented. The inclusion criteria were persons 18 years and older, with varying socio-economic backgrounds and health statuses, who accessed the university wellness services stations in the four communities. For an unknown population with an unknown prevalence of oral health problems (50%), with a confidence level of 95% and a margin of error of ± 5%, a sample of 384 was calculated. A convenience sample of at least 100 respondents per site (*N* = 400) was targeted.

### Instrument

A researcher-administered paper-based questionnaire, which included validated scales with established validity and reliability was used. This questionnaire comprised four sections, namely Section A, Section B, Section C and Section D. Section A included four questions related to respondents’ socio-demographic characteristics. Section B included the WHO Oral Health Questionnaire for Adults,^17^ consisting of 13 questions associated with respondents’ oral health status and practices. Section C consisted of the Oral Health Knowledge Inventory developed by Rustvold^22^ and included 10 questions related to respondents’ oral health knowledge. These questions were adapted from multiple-choice questions to True or False questions to more accurately reflect the anticipated knowledge level of community members and to simplify response options for better comprehension. Section D included 14 questions developed by Rustvold^22^ to measure respondents’ oral health attitudes. All questions were previously validated and applied within the study context.^23^ The questionnaire was translated into Afrikaans and isiXhosa by a professional translator using the forward–backward translation method. It was subsequently pilot tested with a small sample of respondents to identify any challenges in its administration. The internal consistency of the questionnaire was tested using Cronbach’s alpha test with an acceptable value of 0.722 for ‘Knowledge’, 0.579 for ‘Attitudes’ 0.579 and 0.866 for ‘Practices’.

### Data collection procedures

Data were collected in March 2024 by fourth-year nursing students who were fluent in the local respondents’ language. To ensure consistency and data quality, the research team provided training to students on survey administration and supervised the students throughout the data collection process. Safe and accessible data collection venues, such as community halls or gazebos, chairs, and tables, were pre-arranged with the community leaders. Data were collected at different stations, including an explanation about the study to the respondents and distribution of consent forms (Station 1) and completion of the questionnaires (Station 2). The completion of the questionnaires took approximately 20 min. Once completed, all questionnaires were reviewed by the research supervisors for accuracy and completeness.

### Data analysis

Data collected from questionnaires were captured in Research Electronic Data Capture (REDCap; Vanderbilt University Medical Center, Nashiville, TN, United States [US])^24^ and cleaned. The data were imported into the Statistical Package for the Social Sciences (SPSS, version 29) (IBM, Armonk, NY, US) for analysis.^25^ Descriptive statistics, including frequencies, percentages, and means, and standard deviations as applicable, were used to describe respondents’ socio-demographic characteristics, oral health knowledge, attitudes, and practices. Knowledge score was calculated by summing the correct answers and by classifying correct answers as knowledgeable, missing answers as ‘lack of knowledge’, and incorrect answers as ‘misinformed’. Attitudes are reported across three domains, with an overall attitude score calculated by summing positive statements and reversed attitude statements. Individual practices are reported by frequency and percentage. Poor health was coded as ‘Yes’ if respondents self-rated their health as ‘poor’ or ‘very poor’. The Chi-square test was used to determine the association between socio-demographic factors and the practices scale, while the Mann–Whitney U test was used to compare knowledge and attitude scores across categorical variables. Direct logistic regression was performed to assess the impact of several factors on the dependent measure of oral or dental pain reported in the last 12 months. The model contained four independent variables (gender, setting, health status, and positive attitudes) selected based on significance in univariate analysis. Significance was set as *p* < 0.05.

### Ethical considerations

Ethics approval was obtained from the University of the Western Cape Biomedical Research Ethics Committee (BM24/1/2), and permission was obtained from each community leader at the four sites. The study was registered on the National Health Research Database and approved by the Western Cape Department of Health (WC_202403_027). The purpose of the study and respondents’ rights were explained to them. The fundamental research principles, namely, respect for individuals, justice, and beneficence, were considered. Consent forms were available in Afrikaans, English, and isiXhosa and distributed for signing to those who voluntarily agreed to participate in the study. Anonymity, privacy, and confidentiality were ensured. Respondent-identifier details were not collected.

## Results

### Sample characteristics

A total of 776 community members were included in the study; two non-binary respondents identified were excluded from the analysis (*n* = 774). The majority of the respondents were female (*n* = 567, 73.1%). Respondents’ ages ranged from 18 to 85 years, with a mean age of 42.3 ± 14.2 years, and no significant differences between male and female respondents. Nearly half of the respondents (*n* = 365, 46.9%) were from community A, an urban central business district community. Just less than half (*n* = 374, 48.3%) had completed high school or above (Table 1). In terms of their oral health status, nearly two-thirds of the respondents reported that their teeth or mouth caused them discomfort or pain in the last 12 months (*n* = 492, 63.6%), with significantly more female respondents reporting discomfort or pain in the last 12 months (*n* = 379, 63.6% vs. 113, 54.6%, *χ*^2^ = 9.8, *p* = 0.002) (Table 1).

**TABLE 1:** Demographics and oral health status (*N* = 774).

Questions	Male (*n* = 207)		Female (*n* = 567)		Total (*n* = 774)†		Test *χ*^2^	*p*-value
	*n*	%	*n*	%	*n*	%		
**Study site**	-	-	-	-	-	-	13.3	0.004
A (urban)	87	42.0	276	48.70	363	46.9	-	-
B (semi-rural)	26	12.6	109	19.20	135	17.4	-	-
C (semi-rural)	20	9.7	44	7.80	64	8.3	-	-
D (urban)	74	35.7	138	24.30	212	27.4	-	-
**Highest level of study**	-	-	-	-	-	-	1.3	0.253
< High school	114	55.1	287	50.40	400	51.7	-	-
≥ High school	93	44.9	282	49.60	375	48.3	-	-
**Poor health status**	40	19.3	135	23.68	174	22.5	1.6	0.187
**Oral health status**	-	-	-	-	-	-	9.8	0.002
Pain or discomfort in the last 12 months	113	54.6	379	66.80	492	63.6	-	-

† , Two non-binary respondents excluded from the analysis.

### Oral health knowledge

Oral health knowledge was measured using 10 True or False statements. The mean knowledge score was 6.84 ± 1.4 (68.4%) out of 10, with no significant differences between male and female respondents. However, respondents with a high school or higher education level had significantly higher knowledge scores (7.02/10 ± 1.4 vs. 6.68/10 ± 1.4, respectively, *U* = 3.1, *p* = 0.002) (not shown in Table 2).

**TABLE 2:** Oral health knowledge (*N* = 774).

Knowledge-related question responses	Knowledgeable		Misinformed		Lack of knowledge	
	*n*	%	*n*	%	*n*	%
**Domain: Knowledge about oral health practices**						
The goal of brushing our teeth is to remove food and bacteria from all tooth surfaces (True)	737	95.2	27	3.5	10	1.3
Fluoride in toothpaste helps in reducing holes in teeth (cavities) (True)	539	69.6	147	19	88	11.4
The two most important dental habits are brushing once a day and flossing (cleaning between teeth) once a week (False)	415	53.6	306	39.5	53	6.8
You should stop flossing (cleaning between teeth) if your gums start to bleed a little (False)	165	21.3	551	71.2	58	7.5
**Domain: Knowledge about oral health risk factors**						
Smoking can cause gum disease (True)	688	88.9	41	5.3	45	5.8
Sugar harms your tooth surface (enamel) (True)	648	83.7	78	10.1	48	6.2
It is best to have a sugary treat with a meal (True)	194	25.1	543	70.2	37	4.8
**Domain: Knowledge of causes of periodontal disease**						
Plaque is a substance that contains germs and collects on the surface of teeth (True)	676	87.3	44	5.7	54	7
**Domain: Knowledge about periodontal disease**						
Gingivitis (gum inflammation) is when gums become swollen and bleed (True)	628	81.1	73	9.4	73	9.4
**Domain: General knowledge about oral health**						
There is no connection between your oral (mouth) health and your general (body) health (False)	428	55.3	280	36.2	66	8.5

Respondents were most knowledgeable about *the goal of brushing our teeth is to remove food and bacteria from all tooth surfaces*, with nearly all respondents correctly rating this statement as True (*n* = 737, 95.2%) (Table 2). Respondents were most misinformed about *it is best to have a sugary treat with a meal*, with 543 (70.2%) rating this item (False) incorrectly and *you should stop flossing* (cleaning *between teeth*) *if your gums start to bleed a little*, with 551 (71.2%) rating this item (False) incorrectly. Around 10% of respondents were uninformed that *gingivitis (gum inflammation) is when gums become swollen and bleed*, with 73 (9.4%) rating this item (True), and *fluoride in toothpaste helps in reducing holes in teeth* (*cavities*), with 88 (11.4%) rating this item (True) (Table 2).

### Oral health attitudes

The mean attitude score was 39.7 (standard deviation [s.d.] ± 5.4) out of a possible 56, with observed scores ranging from 26 to 55. There were significant differences in attitude scores between semi-rural and urban sites, with participants from urban areas demonstrating significantly more positive attitudes (40.3 ± 5.3 vs. 37.9 ± 5.3; *U* = 5.4, *p* < 0.001). Similarly, respondents with a high school education or higher demonstrated significantly more positive attitudes compared to those with less than a high school education (40.9 ± 5.1 vs. 38.6 ± 5.6; *U* = 5.9, *p* < 0.001). Respondents with pain and discomfort in the last 12 months had significantly lower attitude scores (39.3, ± 5.5 vs. 40.4, ± 5.4, *U* = 2.5, *p* = 0.011) (not shown in Table 1).

In attitude statements on positive beliefs on internal locus of control and self-efficacy, more than 80% of the respondents agreed with all five statements, with the highest level of agreement being for *Knowledge about dental health could help prevent the loss of my teeth* (*n* = 724, 93.5%) (Table 3).

**TABLE 3:** Oral health attitudes (*N* = 774).

Internal locus of control and self-efficacy	Agree		Disagree	
	*n*	%	*n*	%
Knowledge about dental health could help prevent the loss of my teeth	724	93.5	-	-
I believe I know how to brush my teeth correctly	709	91.6	-	-
I believe that I am responsible for preventing the loss of my teeth	708	91.5	-	-
I believe that by brushing and flossing my teeth, I am less likely to get tooth decay	632	81.7	-	-
I believe that by flossing (cleaning between) my teeth, I can prevent gingivitis (gum inflammation)	629	81.3	-	-
**Beliefs about barriers**				
I believe that if my parents have bad teeth, brushing and flossing will not help my teeth	-	-	551	71.2
I believe visiting the dentist is only necessary when I am experiencing pain	-	-	444	57.4
I believe dentures are less troublesome than taking care of my natural teeth	-	-	434	56.1
I believe that only the dentist can prevent cavities	-	-	313	40
If my gums bleed when I brush, this usually means that I am brushing too hard, and I should stop brushing my teeth	-	-	308	39.8
If my gums bleed when I floss, this usually means that I am hurting my gums, and I should stop flossing my teeth	-	-	194	25.1
**Beliefs on susceptibility**				
I am likely to have gingivitis or gum disease in the next year or two	-	-	437	56.5
I am likely to have tooth decay in the next year or two	-	-	376	48.6
I believe that tooth loss is a normal part of growing old	-	-	237	30.6

There were mixed beliefs about barriers related to an external locus of control, low self-efficacy, and negative beliefs about susceptibility. High levels of disagreement, indicating positive attitudes towards barriers ranged from nearly three-quarters of the respondents (*n* = 551, 71.2%) disagreeing with the statement *I believe that if my parents have bad teeth, brushing and flossing will not help my teeth* to only a quarter (*n* = 194, 25.1%) disagreeing with the statement *If my gums bleed when I floss this usually means that I am hurting my gums and I should stop flossing my teeth* (Table 3).

### Oral health practices

Oral health practices were measured through teeth cleaning practices, dental visits and habits around substances that can damage teeth.

#### Teeth cleaning practices

Almost two-thirds of respondents reported brushing their teeth two or more times per day (*n* = 475, 61.4%), while a further 264 (34.1%) reported brushing once daily. Most respondents used a toothbrush (*n* = 756, 97.7%) and toothpaste (*n* = 756, 97.7%), with 514 (66.4%) stating that their toothpaste contains fluoride. However, only 107 (13.8%) of the respondents reported using floss to clean their teeth, with significant differences between semi-rural and urban respondents (*n* = 19, 9.5% vs. *n* = 88, 15.3%, *χ*^2^ = 4.1, *p* < 0.043), and level of education (*n* = 44, 11% less than high school vs. *n* = 63, 16.8% with high school or higher, *χ*^2^ = 5.5, *p* < 0.019).

#### Dental visits

The WHO’s recommendation^17^ is to see a dentist at least once a year for a check-up and professional cleaning. Only 149 (19.3%) respondents visited the dentist in the last year, 517 (66.8%) sometime in the last 5 years, and 108 (14%) respondents had never visited a dentist. There were significant differences in visits to dentists, with fewer visits in the last year among semi-rural respondents than among urban respondents (*n* = 25, 12.6% vs. *n* = 124, 21.6%, *χ*^2^ = 8.6, *p* = 0.013). Similarly, fewer visits in the last year for respondents with less than high school education compared to those with high school education or higher (*n* = 59, 14.8% vs. *n* = 90, 24.1%, *χ*^2^ = 17.1, *p* < 0.001).

Of the respondents who visited a dentist in the last year (*n* = 149), more than half (*n* = 87, 58.4%) were for pain or trouble with teeth, gums, or mouth, and only 17 (11.4%) went for a routine check-up. Similarly, among respondents who visited a dentist in the last 5 years, 341 (66%) did so for pain or trouble with teeth, gums, or mouth, and only 35 (6.8%) went for a routine check-up.

#### Intake of substances harmful to teeth

Over two-thirds of the respondents reported having sugar with their tea or coffee every day or several times a day (*n* = 504, 65.1%), followed by soft drinks with sugar (*n* = 343, 44.3%) and sugary gum or sweets (*n* = 324, 41.9%). Daily consumption of sugar in tea or coffee differed significantly by education level, with respondents who had less than a high school education reporting higher intake compared to those with a high school education or higher (65.6%, *n* = 242 vs. 55.9%, *n* = 203; *χ*^2^ = 7.1, *p* = 0.007). Respondents who consumed soft drinks with sugar daily reported significantly more discomfort or pain in their teeth or mouth in the last 12 months (*n* = 235, 47.8% vs. *n* = 108, 38.3%, *χ*^2^ = 6.5, *p* = 0.011).

Although consuming fruit is recommended, frequent consumption, especially acidic fruits and juices, may increase the risk of dental erosion and caries because of their acidity and sugar content.^26^ Almost half of the respondents consumed fruit every day or several times a day (*n* = 322, 41.6%), with significant differences between semi-rural and urban respondents (*n* = 59, 29.6% vs. *n* = 263, 45.7%, *χ*^2^ = 15.7, *p* < 0.001) and level of education (less than high school *n* = 140, 35% vs. *n* = 182, 48.7% high school education or higher, *χ*^2^ = 14.8, *p* < 0.001). Confectionery such as biscuits or pies was eaten every day or several times a day by 150 (19.4%) of the respondents, with no significant differences among groups.

Nearly a third (*n* = 249, 32.2%) smoked cigarettes every day, and 254 (34.1%) drank two or more drinks of alcohol daily in the past 30 days. There were significant differences in smoking and drinking more than two drinks per day. More male respondents than female respondents reported drinking more than two drinks a day (*n* = 96, 46.4% vs. *n* = 158, 27.9%, *χ*^2^ = 23.6, *p* < 0.001).

#### Prediction of oral or dental pain in the last 12 months

A multivariable binary logistic regression was performed to identify factors associated with the dependent variable, *reported oral or dental pain in the last 12 months*. The model contained four independent variables (gender odds ratio [OR] [female] = 1.7 [95% confidence interval {CI}: 1.2–2.3]), setting (OR semi-rural = 2.3 [95% CI: 1.6–3.4]), health status poor (OR = 1.9 [95% CI: 1.3–2.9]), and positive attitudes (OR = 0.96 [95% CI: 0.93–0.99]). The full model containing all predictors was statistically significant, *χ*^2^(4, *N* = 774) = 55.7, *p* < 0.001, indicating that the model was able to distinguish between the cases. Only three variables made a unique statistically significant contribution to the model (self-reported oral or dental pain in the last 12 months): gender (female 66.8% vs. male 54.6%), setting (semi-rural 76.9% vs. urban 59.0%), and self-rated health (poor 74.7% vs. good 60.3%). Respondents residing in semi-rural areas had higher odds of reporting oral or dental pain in the last 12 months compared to those in urban areas (adjusted odds ratio [aOR]: 2.1; 95% CI: 1.4–3.1) (Table 4).

**TABLE 4:** Prediction of self-reported oral or dental pain in the last 12 months.

Variable	B	Significance	aOR	95% CI
Gender (female)	0.492	0.004	1.6	1.2–2.3
Setting (semi-rural)	0.746	< 0.001	2.1	1.4–3.1
Self-rated health (poor)	0.577	0.004	1.8	1.2–2.7
Positive attitudes	−0.166	0.120	0.9	0.6–1.1

CI, confidence interval; aOR, adjusted odds ratio.

## Discussion

This study examined oral health knowledge, attitudes, and practices in two urban and two semi-rural communities in the Western Cape, South Africa. Overall, respondents showed a moderate level of oral health knowledge, positive but inconsistent attitudes, and varied oral health practices. Significant differences were observed based on education level and place of residence, but not by gender. Regression analysis identified rurality as a significant predictor of oral or dental pain, with participants from semi-rural areas being 1.5 to 3.5 times more likely to report pain. Notably, 63.6% of respondents reported experiencing oral or dental pain or discomfort in the past 12 months. This finding aligns with a 2020 study conducted in Bellville, Western Cape, which reported that 68% of adults had high levels of decayed and missing teeth.^20^

### Knowledge

Respondents demonstrated good knowledge regarding some basic oral health concepts. Almost all the respondents (95.2%) recognised that the purpose of toothbrushing is to remove food debris and bacteria, while more than four out of five correctly identified plaque as a bacterial deposit on the tooth surface. Similarly, almost 70% acknowledged the role of fluoride in preventing dental caries. These findings are consistent with a 2021 study in South India that found that 84% of adult respondents living in a residential community could correctly define plaque and 75% understood the protective role of fluoride toothpaste.^27^ In Rwanda, Nzabonimana et al. also reported that more than 82% of adults recognised the importance of interdental cleaning for preventing gum inflammation.^28^ However, in this study, only just over half of the respondents (53.6%) demonstrated knowledge about interdental cleaning, and the majority (70.2%) were misinformed about the appropriate timing of sugar consumption. These gaps are consistent with findings from Nigeria, where Akinyamoju et al. reported that fewer than half of street vendors recognised the link between sugar consumption and dental caries.^29^ Importantly, respondents with higher education levels in our study consistently demonstrated better knowledge, echoing findings from China, where Zhao et al. reported that adults with lower education were at significantly higher risk of poor oral health outcomes, despite a general lack of periodontal disease knowledge even among groups with higher levels of education.^30^ This pattern highlights the central role of education in shaping oral health awareness and behaviours.

### Attitudes

The overall attitudes of respondents towards oral health were moderately positive. Respondents from semi-rural areas, lower levels of education, and those who experienced pain or discomfort in the last 12 months had significantly more negative attitudes. There was a high degree of agreement with statements emphasising personal oral hygiene practices, suggesting an internal locus of control and awareness of self-care. However, 60% of respondents believed that only dentists could prevent cavities and almost 70% believed that tooth loss is a normal part of growing old. This is comparable to findings from India, where Kumar et al. reported that 61% of patients believed dentists focus primarily on treatment rather than prevention, and fewer than half sought dental care except when experiencing pain.^31^ Theories of reasoned action and planned behaviour suggest that such attitudes shape practices and that limited appreciation for prevention and susceptibility likely contributes to delayed health-seeking behaviours.^32^

### Practices

Brushing habits in this study were generally good, with most respondents brushing twice daily with fluoridated toothpaste. This finding is consistent with another South African study in Manguang, where Modikoe et al. reported that all respondents brushed daily, although with less consistency regarding frequency.^33^ By contrast, Kumar et al. found that in India only 35% of respondents brushed twice daily, and dental floss use was negligible.^31^ In this study, flossing was notably limited, with fewer than 15% reporting regular use. This aligns with findings from a 2024 study in Rwanda, where most adult respondents (92.7%) attending public health facilities had never used dental floss to clean their teeth.^28^ Similarly, in China, Zhao et al. reported that 90% of the 50 991 respondents had never flossed their teeth.^30^ Conversely, over 60% of respondents in another South African study reported daily flossing.^33^ These disparities highlight how contextual, cultural, and systemic factors shape practices. Dental visits in this study were largely problem-driven, with fewer than a quarter attending for preventive services. A similar trend was reported in Rwanda, where nearly 80% sought dental care only when experiencing pain, reinforcing the reactive rather than preventative orientation to oral health-seeking behaviours in many low- and middle-income settings.^28^ Respondents from semi-rural areas were less likely to visit the dentist, indicating limited access to such services, as neither of the two communities had dental services available. Lastly, high levels of sugar consumption in tea, coffee, and fruit intake were observed in this study, raising concern about preventable dietary risks for caries and periodontal disease. While no gender differences were observed in knowledge or attitudes in this study, other research has reported that women demonstrate better oral health awareness and practices,^6^ which may reflect broader socialisation roles and caregiving responsibilities. See Appendix 1 for oral health practices key findings.

### Implications

The findings highlight persistent knowledge gaps and suboptimal practices, despite generally positive attitudes. Education level emerged as a key factor influencing oral health knowledge and practices, underscoring the need for targeted health promotion strategies across the lifespan. In South Africa, while oral health may be addressed at a primary school level, the responsibility of continued learning shifts to individuals, families, and communities. This creates missed opportunities for reinforcement, particularly among those with limited access to formal education.

### Recommendations

Considering these findings and the high prevalence of oral health diseases, health professionals, particularly oral hygienists (if available) and primary care nurses, play a pivotal role in strengthening health literacy during clinical encounters. Health professional training programmes should emphasise oral health education, as most periodontal conditions are preventable. Expanding preventative messaging and improving access to tailored preventive services could significantly reduce the oral health burden, especially in semi-rural and rural communities.^34^ We recommend that the study should be replicated in other settings beyond Cape Town, South Africa, and could be adapted into a longitudinal design to assess changes in oral health knowledge, attitudes, and practices over time and evaluate the impact of targeted interventions.

### Limitations

While this study provides interesting insights about the knowledge, attitudes, and practices of community members in the Western Cape, the results cannot be generalised to the wider South African population, as demographics, oral health behaviours and access to care may differ across provinces. The sample was also convenient and predominantly female, 73.1% compared with 51.7% reported in the City of Cape Town population,^21^ which may limit representativeness.

## Conclusion

Adults in urban and semi-rural communities in the Western Cape demonstrated good knowledge on some basic concepts of oral health and generally positive attitudes; however, important gaps persist in areas such as sugar consumption, flossing, and preventive dental visits. Education was a key factor influencing knowledge and practices, highlighting the need for targeted health promotion. Furthermore, rurality was a determinant of self-reported oral or dental pain. Strengthening oral health education, increasing oral health community outreach, integrating preventive counselling into routine care, and empowering health professionals, especially primary care nurses, to reinforce oral hygiene practices are essential to reduce preventable periodontal disease and improve oral health outcomes.

## Figures and Tables

**FIGURE 1-A1:**
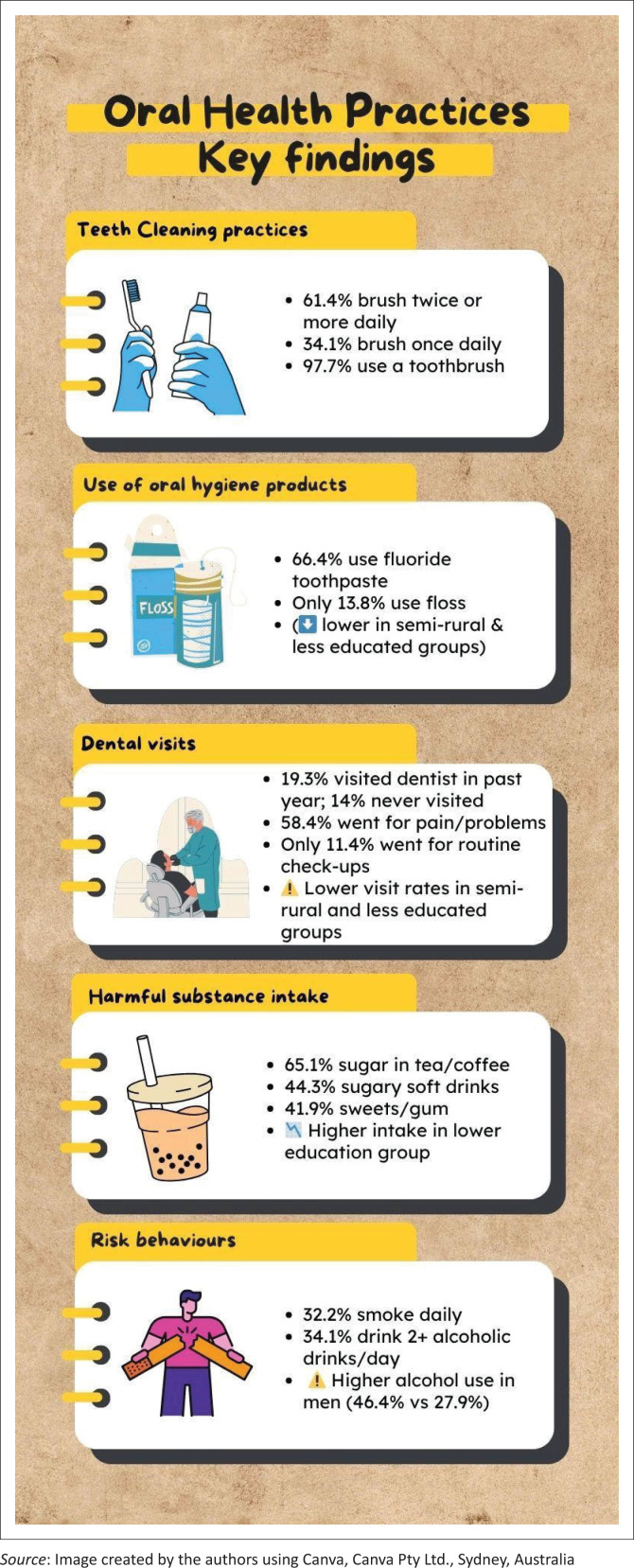
Key findings on oral health practices.
